# A historical overview of the left ventricular reconstruction

**DOI:** 10.1186/1749-8090-8-S1-O131

**Published:** 2013-09-11

**Authors:** H Suma

**Affiliations:** 1 Suma Heart Clinic, Tokyo, Japan

## 

Over the past 3 decades, several observational studies established a role for the left ventricular reconstruction procedure (LVR) in the treatment of ischemic cardiomyopathy. In 2009, the Surgical Treatment for Ischemic Heart Failure (STICH) trial reported their findings and found no benefit of adding LVR to coronary artery bypass surgery in ischemic dilated cardiomyopathy. The STICH findings precipitated a decline in interest in LVR. In the presentation, I review the historical background and observational data that established a role for LVR. The STICH trial should be contended that the limitations are such that the study cannot provide any reliable conclusion on the role of SVR because of suboptimal patient selection and inadequacy of volume reduction (only19% mean reduction in volume). Several post-STICH publications continue to demonstrate that LVR is effective in dilated ventricles, provided the procedure achieves >30% volume reduction. It is critical that surgeons continue their work in LVR, and continue to analyze their data, to enable better clarification of the indications and future role for this procedure.

